# Clinical reasoning pattern used in oral health problem solving – A case study in Indonesian undergraduate dental students

**DOI:** 10.1186/s12909-022-03808-7

**Published:** 2023-01-23

**Authors:** B. E. Chrismawaty, O. Emilia, G. R. Rahayu, I. D. Ana

**Affiliations:** Department of Oral Medicine, Faculty of Dentistry, Universitas Gadjah Mada, Jl. Denta 1, Sekip Utara, Yogyakarta 55281, Indonesia

**Keywords:** Clinical reasoning, Knowledge structure, Undergraduate dental student, Oral health problem solving, Hypothetical clinical case, Concept map, SOLO taxonomy

## Abstract

**Background:**

Health professionals are known to use various combinations of knowledge and skills, such as critical thinking, clinical reasoning, clinical judgment, problem-solving, and decision-making, in conducting clinical practice. Clinical reasoning development is influenced by knowledge and experience, the more knowledge and experience, the more sophisticated clinical reasoning will be. However, clinical reasoning research in dentistry shows varying results .

**Aims:**

This study aims to observe the clinical reasoning pattern of undergraduate dental students when solving oral health problems, and their accordance with their knowledge acquisition.

**Material and methods:**

This qualitative study employed the think-aloud method and the result was assessed through verbal protocol analyses. Five respondents from final year dental undergraduate students were agreed to participate. A unique hypothetical clinical scenario was used as a trigger. The audio data were transcribed, interpreted, and categorized as a clinical reasoning pattern; and the concept maps created were assessed by a Structure of Learning Outcomes (SOLO) taxonomy as knowledge acquisition.

**Results:**

Observations on clinical reasoning patterns and the level of knowledge acquisition in five undergraduate dental students showed varying results. They applied clinical reasoning patterns according to their knowledge acquisition during didactical phase. Learners with inadequate knowledge relied on guessing, meanwhile learners with adequate knowledge applied more sophisticated reasoning pattern when solving problems.

**Conclusions:**

Various problem-solving strategies were encountered in this study, which corresponded to the level of knowledge acquisition. Dental institutions must set minimum standards regarding the acquisition of conceptual knowledge accompanied by improvement of clinical reasoning skills, as well as refinement of knowledge and procedural skills.

**Supplementary Information:**

The online version contains supplementary material available at 10.1186/s12909-022-03808-7.

## Introduction

Clinical reasoning is an essential component of health care professional practice [1, 2]. It can be defined as a skill, process, or outcome [3], which enables clinicians to identify, collect and process information, determine diagnoses, and provide accurate decisions regarding treatment options [4–6]. Diagnostic errors can be minimized by implementing appropriate clinical reasoning strategies, which, in turn, affect the improvement of patient outcomes and safety [7]. Research regarding the development and assessment of clinical reasoning has long been conducted in the medical profession [8, 9], followed by nursing and related health professions. These various studies revealed that every healthcare professional has distinctive and unique clinical reasoning when solving problems and making clinical decisions [10–12]. Clinical reasoning is used and applied in accordance with the problem space of each profession [13–15].

Clinical reasoning pattern in dentistry corresponds to dental environment, where the primary care situation requires dentists to provide fast but effective treatment. Generally, the main action is the treatment of teeth and their supporting tissues disease. When making clinical decisions, it is common to choose between alternative treatments, such as dental crowns versus fillings or tooth extraction versus root canal treatment [16–20]. Due to similarity in clinical problems, dentists generally apply “intuition” or “pattern recognition” in solving problems [1, 16, 19, 21, 22]. The “pattern recognition” approach helps solve clinical problems, when dental care services are extraction, restoration or preventive based treatments. However, when dental care services shifts to diagnosis-based, this reasoning approach can complicate clinical problem solving [23]. This is related to complexity clinical problems in dentistry, which can involve various complicating factors in determining diagnosis and treatment, such as multiple systemic diseases and medications. Thus, the considerations in establishing treatment become increasingly complex as they involve patient safety and well-being [5, 24–26].

Referring to the theory of medical expertise, “pattern recognition” approach is not yet available or exist in undergraduate dental students as the lowest expertise level or novices. This reasoning approach requires restructuring of knowledge and repeated clinical exposure or experience [13, 27, 28]. The application of this reasoning pattern by novices with limited knowledge structure and clinical experience is prone to diagnostic errors [29].

## Background

### Definition and paradigm of clinical reasoning

Clinical reasoning is strategy of thinking and decision-making processes, and considered to foster clinical practice and competence [30, 31]. Clinical reasoning varies across healthcare professions, because it encompasses a set of problem spaces, which is defined by the unique framework of reference, workplace, practice model and patient context of the clinician [32]. Clinical reasoning involves the following: prudent action in the sense of taking the best course of action according to a specific context; professional action in making decisions, demonstrating ethical, accountable and self-regulatory behavior; person-centered who shows respect and can collaborate with clients, caregivers, and colleagues [1]. Every health professional engages in clinical reasoning by gathering and synthesizing clinical evidence, generating hypotheses, formulating an impression and decisive prognosis and diagnosis, and eventually determining treatment, care, and/or management plan [2, 29, 33, 34]. Clinical reasoning in medical practice is currently dominated by two theoretical frameworks, namely the process and structural paradigm. The process paradigm comprises cognitive processes involved in clinical decision making and the structural paradigm is connected to knowledge storage and recalls from memory [35, 36].

#### Process paradigm of clinical reasoning

The cognitive processes involved in the clinical reasoning paradigm emphasized on clinical judgment as intuitive and analytical approach [29, 37]. The cognitive process of clinical reasoning varied involves the following: hypothetico-deductive [38], elaborated hypothetico-deductive [39], heuristic reasoning [40], schema-induction reasoning [41, 42], and pattern recognition [33, 43]. The reasoning process can be determined by analyzing the content of transcription result from think aloud methods and contrasting and comparing with known clinical reasoning pattern [44–46].

#### Structural paradigm of clinical reasoning

As a structural paradigm, clinical reasoning relies on how clinicians acquire, process, store, and use medical knowledge to solve problems and make clinical decisions [47]. The development of clinical reasoning lies on conceptual and procedural knowledge and is honed with clinical experience [48–50]. Previous studies have shown that problem solving, judgment, and clinical decision making are entrenched in how previously acquired knowledge is obtained, stored, and recalled [39, 49, 51].

### Knowledge acquisition assessment

The knowledge structure in memory can be measured by an assessment [52], concept or conduct test [53], or knowledge survey [54]. Knowledge structure based on its acquisition can be assessed in a formatively or summative. Formative assessment is intended to guide upcoming learning, provide reassurance, promote reflection, and shape values, while summative assessment refers to a comprehensive valuation of competence, suitability for practice, or qualifications for progress to a higher level of responsibility [52]. Concept or conduct test are methods used to determine the ability of students to complete certain tasks or demonstrate mastery of a skill or knowledge of content [53]. Knowledge surveys consist of a series of questions that cover the full content of a course [54].

One of concept test is the structure of learning outcomes (SOLO) taxonomy [55, 56], which was originally developed by Biggs and Collis in 1982 as a content independent taxonomy which offers criteria to categorize the levels of students’ performance when mastering new learning [57]. It could provide descriptions of the structural organization of knowledge. Wood [58] reported that SOLO taxonomy is one of the most popular systems for classifying the structural complexity of students’ knowledge. The level of knowledge described in this taxonomy is not content-specific and can be applied to any stage of learning. The SOLO taxonomy differentiates levels and content of knowledge as follows: P (prestructural), U (unistructural), M (multistructural), R (relational) and Extended Abstract [55–58]. The level of knowledge is categorized as pre-structural if it refers to the use irrelevant information or no meaningful answer; unistructural if the answer manifests a single fact obtain directly from the problem. Multistructural shows a partial understanding by directing on several relevant aspect, but not coordinated yet. Relational knowledge refers to the integration of several relevant information into a coherent whole or have an adequate knowledge related to the topic. The extended abstract of knowledge reached when they able to generalize knowledge beyond the particular problem to be solved, thus knowledge insight has been expanded [55–58].

Concept mapping is a tool that supports the visualization of learning and the manipulation of information. This tool has been used to understand how learning changes a learner’s cognitive structure of knowledge, and support the development of clinical expertise [59, 60]. Expertise has the highest level of knowledge structures, which describes as being elaborate, holistic and highly integrated. This knowledge structure emerged gradually through various structural changes [59]. Boulton-Lewis [56], stated that SOLO taxonomy can serve as a means to develop and assess higher order thinking based on the organizational structure of knowledge in the memory. Boulton [61] observed the written answers and concept maps of student work, showing that the level of knowledge of written answers based on the SOLO taxonomy corresponds to the level of knowledge indicated by the concept map. The use of the SOLO taxonomy regarded as the only assessment method of the extent of a content knowledge in a discipline and its structural organization [56].

## Benefit

The pedagogical stage is the most important basic step in obtaining various conceptual and procedural knowledge in dental education. Knowledge is gradually acquired starting with basic and biomedical science, simple and advanced procedural knowledge and finally enriched with clinical experience. The knowledge structure in memory plays an important role in knowledge retrieval when solving clinical problems. Observation of knowledge structure in the memory of undergraduate dental students can help educators identify errors or inaccuracies in clinical reasoning.

## Aims

This study aims to observe the clinical reasoning patterns of undergraduate dental students when solving oral health problems from hypothetical cases, and their accordance with their knowledge acquisition.

## Material and methods

The research protocol was prepared in accordance to the Declaration of Helsinki on the ethical principles of research involving human subjects, meeting the ethical eligibility from the Medical and Health Research Ethics Committee (MHREC), Faculty of Medicine, Public Health, and Nursing Universitas Gadjah Mada on October, 11 2019 (No: KE/FK/1183/EC/2019). The research is conducted after obtaining approval from the Faculty of Dentistry, Universitas Gadjah Mada, Yogyakarta Indonesia (No.11709/UN1/FKG.1/Set.KGI/ LT/2019). The research was conducted in January 2020. Clinical reasoning ability was determined by verbal protocol analysis with the think aloud method.

### Participants

The research samples were selected purposively, comprising five participants from the final year dental undergraduate students from the Universitas Gadjah Mada, Yogyakarta, Indonesia. The participants were asked to read the aims and methods of the study before recruitment. Only those who signed the informed consent participated in the study. All the participants had already completed their pedagogical phase. All participants are female students from the same entrance year (2015/2016), with ages ranging from 22-23 years old. The academic abilities of the participants varied: three participants were average student with a GPA <3, one participant was good student with a GPA between 3-3.5 and the other was an excellent student with a GPA> 3.5.

### Hypothetical clinical cases

A single hypothetical clinical case was developed as a trigger for clinical problem-solving and decision-making skills. The stability and fidelity of the observed results can be effectively obtained through a single hypothetical clinical case [19]. The clinical findings of a hypothetical case were compiled in a “patient file” format, which was arranged sequentially from the history, clinical examination and additional investigations (see Additional file 1). Several diagnoses can be determined on the basis of this hypothetical clinical case. As the main complaint, sore mouth can lead to mucosal lesions of various form, ranging from atrophic, erosions, fissures to ulceration lesions. Solving the problem will lead to several possible diagnoses, because these oral lesions have various possible etiopathogenesis. Furthermore, one of the most likely diagnoses will lead to clinical decision making, such as treatment or management of oral complaints.

### Data acquisition through verbal or think aloud protocol.

Data acquisition was realized through in-depth interviews guided by semi-structured questions. Observation and data collection were individually performed by previously trained research assistants. Interviews were conducted in a private room away from noise and crowds. The participant was asked to read the instruction carefully. The hypothetical clinical cases were given to the participants, and the research assistant later questioned them based on interviews guidance, which is intended to ensure uniformity of interviews for all participants. Audio recording and notes were made during the interview. The participants were asked to draw a concept map as a summary of their understanding of a hypothetical clinical case at the end of the interview.

### Transcription of audio to text

The recorded data were transcribed verbatim for further analysis through ‘oTranscribe’; a free web-based application designed to obtain of transcribing recorded interviews (https://otranscribe.com/).

### Determination of clinical reasoning pattern

Only few clinical reasoning patterns were used by undergraduate dental students. The most frequently used patterns based on the clinical problem-solving research were hypothetico-deductive, elaborated hypothetico-deductive model, scheme-inductive reasoning, and pattern recognition [1, 22, 30, 43, 62, 63]. The current study applies a predetermined content analysis, with theory or relevant research findings as guidance for initial codes. The transcribed data are thoroughly reviewed and interpreted meticulously to identify clinical reasoning pattern. The model of clinical reasoning from each case study or participant determined as if they fulfilled the accepted definition (Table 1).

**Table 1:** Predetermined content analysis, with theory or relevant research findings as guidance for initial codes of clinical reasoning pattern used in this study [1, 22, 30, 43, 62, 63]

Guessing	Participants are stated to apply guessing when they jump to conclusion, hypotheses or diagnoses after obtaining initial clinical data without considering other additional data nor knowing the relationship between new and initial data.
Hypothetico-deductive reasoning	Participants are stated to apply HD reasoning if they generated several hypotheses or diagnoses during the initial presentation of data, used them to guide further investigation to obtain new information, and later test the hypotheses through reinterpretation data or clarifying new data.
Elaborated model of hypothetico-deductive reasoning	Participants are stated to apply E-HD reasoning if they generated hypotheses as soon as the initial clinical data pieces were available, then collect data. Analysis focused on relevant data. Furthermore, the alleged hypothesis is tested by the process of HD reasoning and consistency evaluated through initial clinical data.
Scheme-inductive) reasoning	Participants are stated to apply scheme-inductive reasoning if they first make a series of specific observations and interpretations of clinical data, determines several suspected diagnoses or hypotheses, and refines it until a diagnostic solution is reached.
Pattern recognition	Participants are stated to apply pattern recognition when determining hypotheses or diagnoses by matching them with information automatically and directly from a well-structured knowledge base.

### Determination of knowledge structure in memory

Concept mapping is composed of nodes, which represent concepts, and the links that connect between concepts. New knowledge is stored constructively by involving previous knowledge; thus, concept mapping is a process of creative activity in which learners exert a conscious effort to explain meaning by identifying key concepts and relationships, and relating them to existing knowledge structures and frameworks [59]. The SOLO taxonomy comprises five levels of sophistication. The lower levels emphasize quantity or the amount of knowledge of a learner, while the higher levels focus on the integration of the details into a structural pattern and the development of relationships between the details along with other concepts outside the learning domain (Table 2).

**Table 2:** Levels and description of the SOLO taxonomy [55, 56, 64], with suggested concept maps (The illustrations depicted are from https://pamhook.com/free-resources/downloadable-resources/)

SOLO taxonomy	Description (the Level of Knowledge Structure)	Suggested map concept
**SOLO 1**: Pre-structural level	The learner does not have a proper understanding of the knowledge or use irrelevant information and / or miss key information.	
**SOLO 2**: Uni-structural level	The learner demonstrates a correct grasp of one aspect, fact or idea obtained directly from the problem and the task to be implemented.	
**SOLO 3**: Multi-structural level	The learner has an incomplete understanding obtained by focusing on several relevant aspects, facts or concepts.	
**SOLO 4**: Relational level	The learner may understand relations between several aspects and how they might fit together to form a whole. The learner responses are robust; they make sense of the various aspects of the topic and integrate the parts into a coherent structure.	
**SOLO 5**: Extended abstract level	The learner may generalize structure beyond the particular problem to be solved, and links the problem to a broad context. The learner may also perceive structure from many different perspectives, and transfer ideas to new areas.	

The SOLO taxonomy can assist educators and researchers systematically to identify levels student performance when mastering new learning taxonomy [55, 56, 64]. The concept maps of the participants were analyzed and categorized in accordance with the different levels of SOLO taxonomy.

## Results

As noted in the title, these articles reported and reviewed five written information from undergraduate dental students when solving a hypothetical case. The transcribed data will be explored and discussed as a case study, one of the qualitative research methods. The case studies can be used to explore a phenomenon in a particular context through various data sources [65]. The phenomenon explored in the current study is the clinical reasoning pattern in the context of oral health problems and diseases. The data source was obtained by verbal protocol through think-aloud method; as a source of information, students had to reveal verbally what was on their mind when asked to solve problems. Therefore, this study examines how students use their structure of knowledge to solve problems. The course of clinical data analysis to the determination of the diagnosis is considered as a clinical reasoning pattern. When presented with clinical problems, the initial response of each participant to clinical data reflected the cognitive ability or knowledge.

The results of some of the thoughts given by the participants showed a significant difference. The think aloud method requires participants to verbalize what they think upon receiving the information written in the script. The interviewer must occasionally lure the participant to further explain the statement. The graph representing their concept map was redrawn by translating it from Bahasa Indonesia to English. The original concept maps prepared by each participant are attached to the Additional file 3.

### Case study 1

**Fig. 1:**
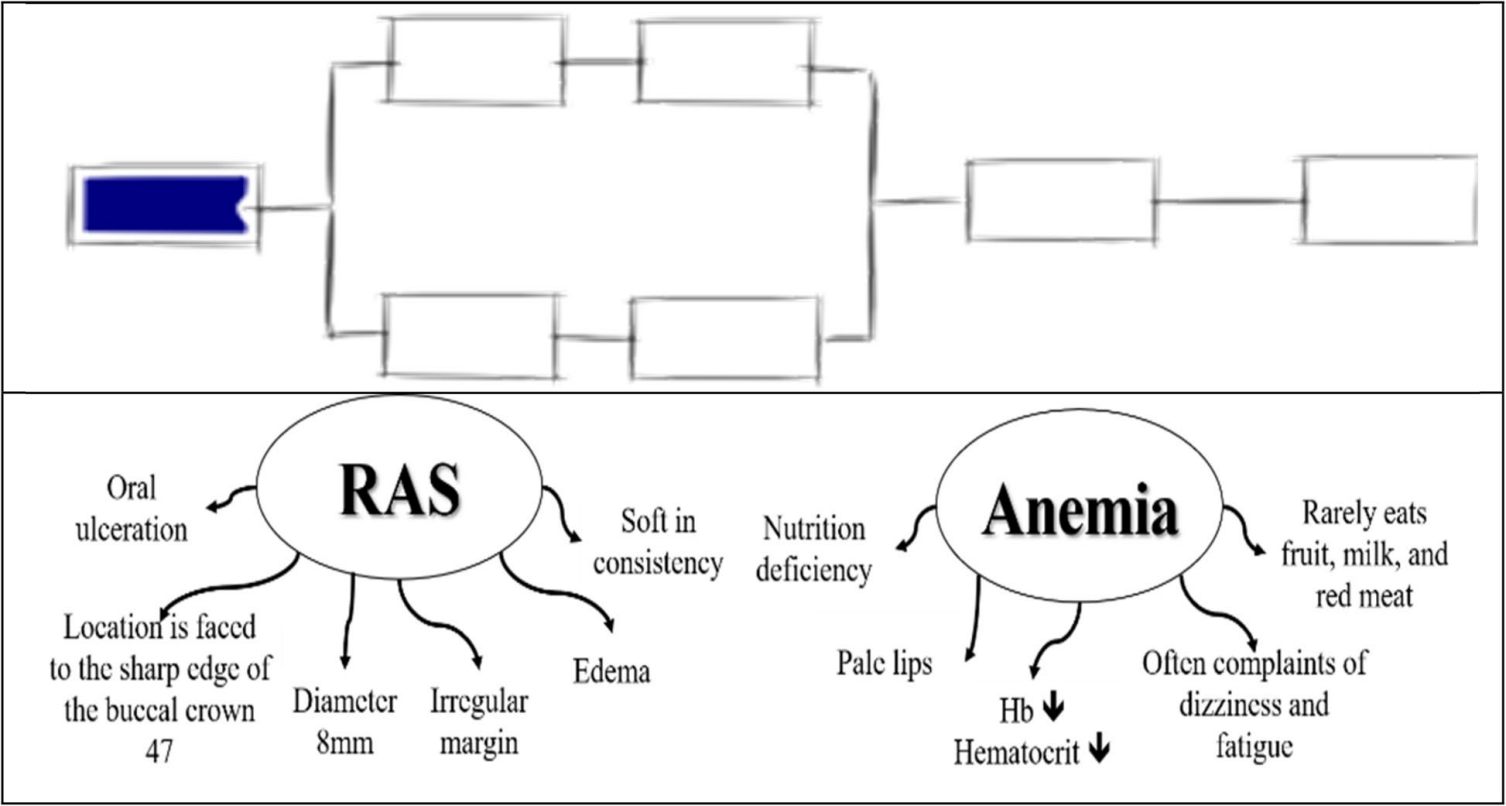
Concept map made by participant #1 shows prestructural level of knowledge (SOLO taxonomy 1). (The illustrations depicted are from https://pamhook.com/free-resources/downloadable-resources/)

Participant #1 took a long time to express their thought in responding to initial clinical information. Participant did not explain the meaning of each clinical information provided, but directly suggested that the patient clinical problem was probably sore mouth, further determining the lesion as recurrent aphthous stomatitis. Participant then explained that this lesion was the most common oral disease encountered in the population. The participant said that the diseases were similar to the complaint of the patient in a hypothetical clinical case during the interview. Participant hesitate or do not know what further information is needed when asked for an explanation. A series of statements from participant #1 in solving problems refers to guessing. The concept map created by participant # 1 showed one or two separate nodes that refer to one aspect, then forms a single chain structure by adding several concepts that are directly related to the main concept but isolated from each other. Participants had enough clinical information but had difficulty recalling relevant knowledge from long-term memory stores, because the knowledge acquired was scattered, disorganized and unstructured in memory. These knowledge level referred to prestructural (SOLO taxonomy 1). (Fig. 1).

### Case study 2

**Fig. 2:**
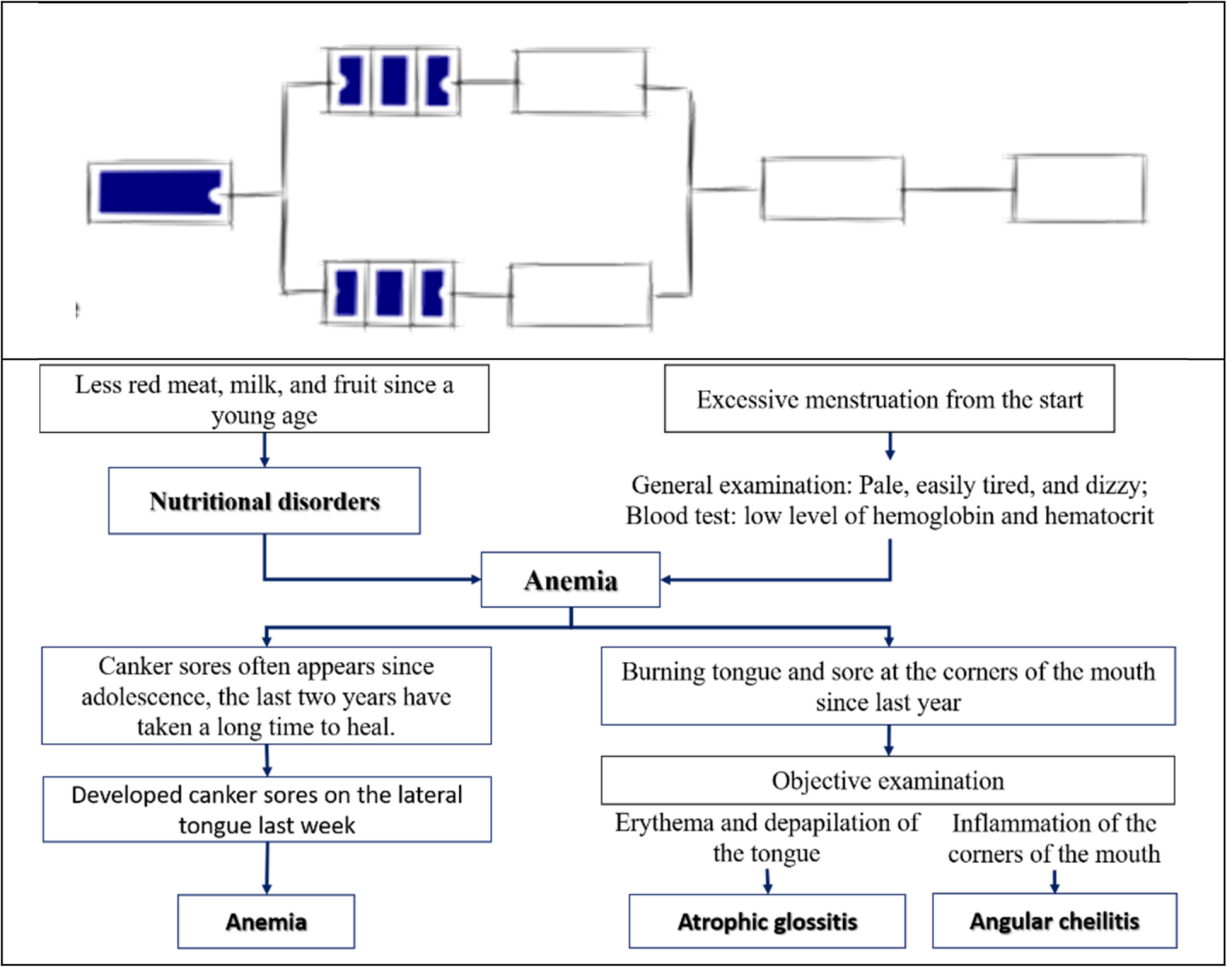
Concept map made by participant #2 showed the multistructural level of knowledge (SOLO taxonomy 3). (The illustrations depicted are from https://pamhook.com/free-resources/downloadable-resources/)

Participant #2 initially stated that they could not decide between hypothesis or definitive diagnosis because the information is incomplete. However, they could determine some hypothesis and even a few suspected diagnoses after the additional information were revealed. The problem-solving strategy by participant #2 refers to a elaborated model of hypothetical-deductive reasoning pattern, which first involves acquisition of clues, formation of hypotheses, interpretation of clues, evaluation of hypotheses. The concept map created by participant # 2 demonstrated a grasp of one clinical fact obtained directly from the scenario, and putting anemia as a focus of problem, and elucidates several oral lesions as consequences (Fig. 2). The concept map built by the participant #2 represents the multistructural level of knowledge (SOLO taxonomy 3). Participants have not been able to coordinate pieces of information that may be related to each other (Fig. 2).

### Case study #3

**Fig. 3:**
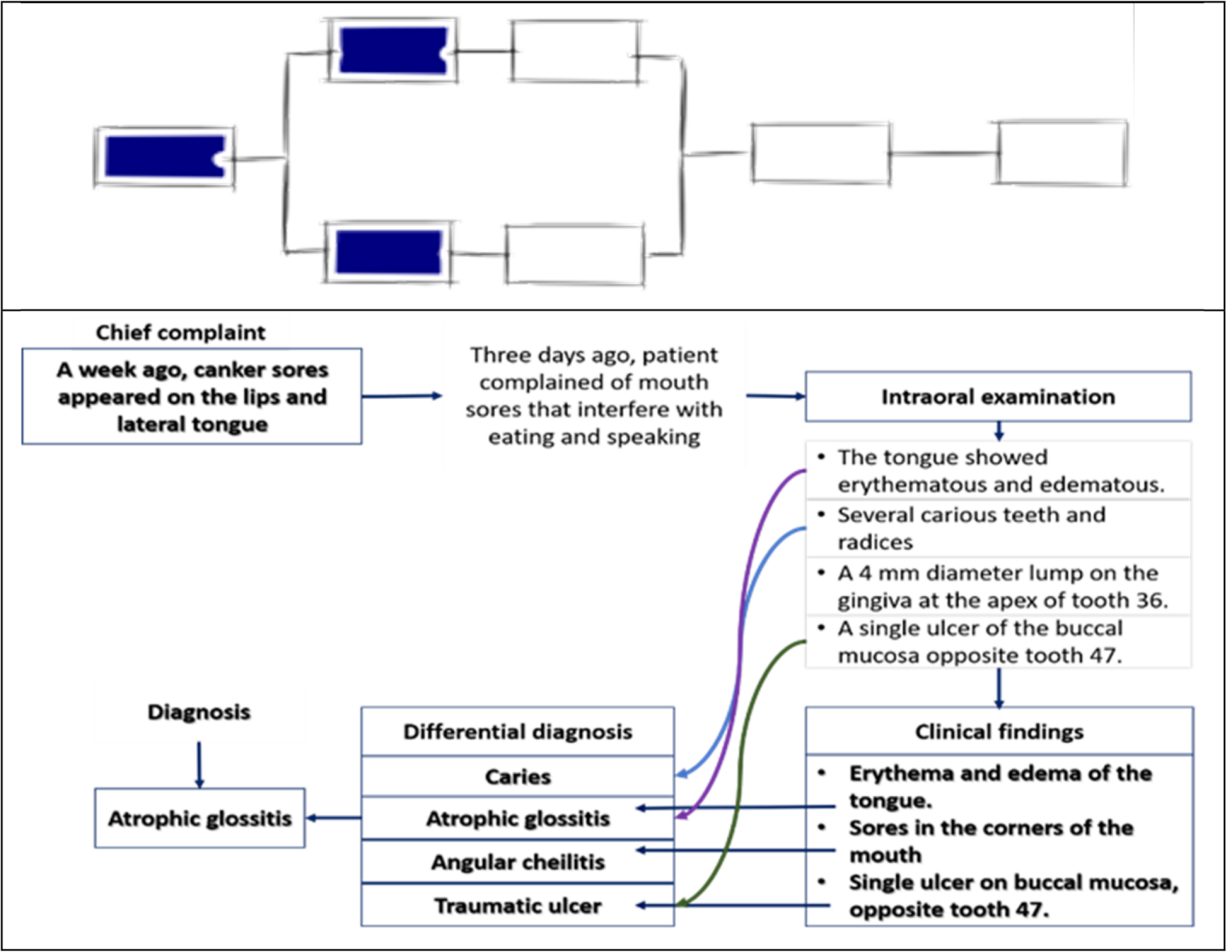
Concept map made by participant #3 showed the unistructural level of knowledge (SOLO taxonomy 2). (The illustrations depicted are from https://pamhook.com/free-resources/downloadable-resources/)

Participant #3 asked for additional data immediately after obtaining the initial information, stating that establishing a hypothesis or diagnosis without that information was difficult. However, the participant established aphthous stomatitis as suspected diagnosis along with similar oral lesions, and attempted to find certain clinical information that can help in confirmation. The applied clinical reasoning pattern is similar to hypothetico-deductive reasoning. The concept map built by participant #3 showed an attempt to correlate the pieces of clinical information from history to clinical findings but were treated separately. This knowledge structure matched to the unistructural level of knowledge (SOLO taxonomy 2) (Fig. 3).

### Case study #4

**Fig. 4:**
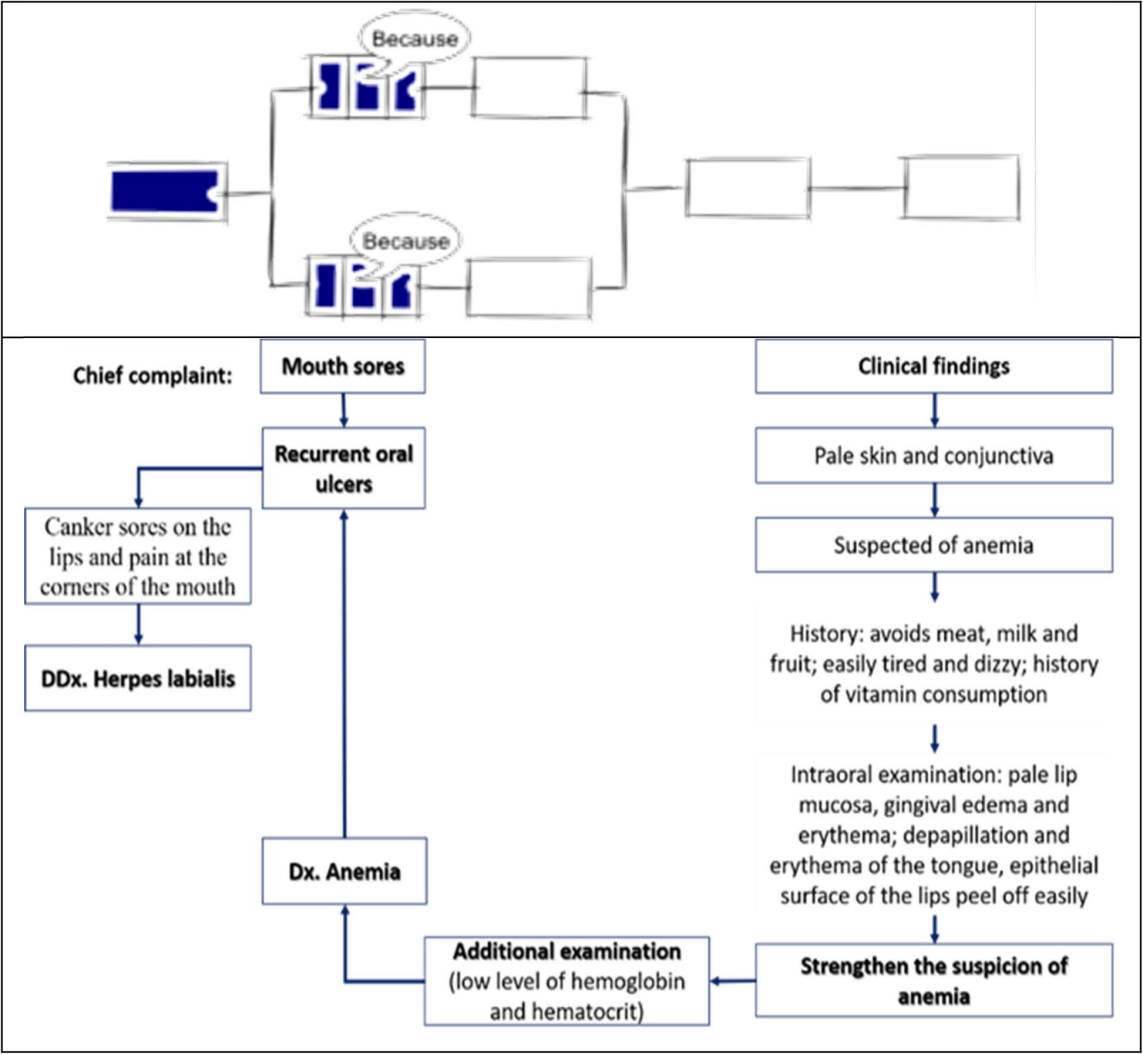
Concept map made by participant #4 showed the relational level of knowledge (SOLO taxonomy 4). (The illustrations depicted are from https://pamhook.com/free-resources/downloadable-resources/)

Participant #4 initially stated that “mouth sore” is the main clinical problem that disturbed oral function, but could not determine the hypothesis or probable diagnosis because the information is still incomplete. The participant then specified aphthous stomatitis as a hypothesis based on the history of present illness, and recognized anemia from additional information as a possible systemic condition responsible for the development of the patient’s complaint. The new information obtained were used to rule out inappropriate hypotheses or add new hypotheses, be combined and analyzed with clinical knowledge acquired to established diagnoses. This problem-solving strategy used refer to forward-driven or inductive schema reasoning pattern. The concept map created reflected the level of knowledge as the relational level (SOLO taxonomy 4), wherein the participants can identify anemia as an excerpt from the medical history and link it to the development of the patient’s complaint. A learner with this level of knowledge structure may understand a relation between several aspects and how they might fit together to form an entire clinical picture (Fig. 4).

### Case study #5

**Fig. 5:**
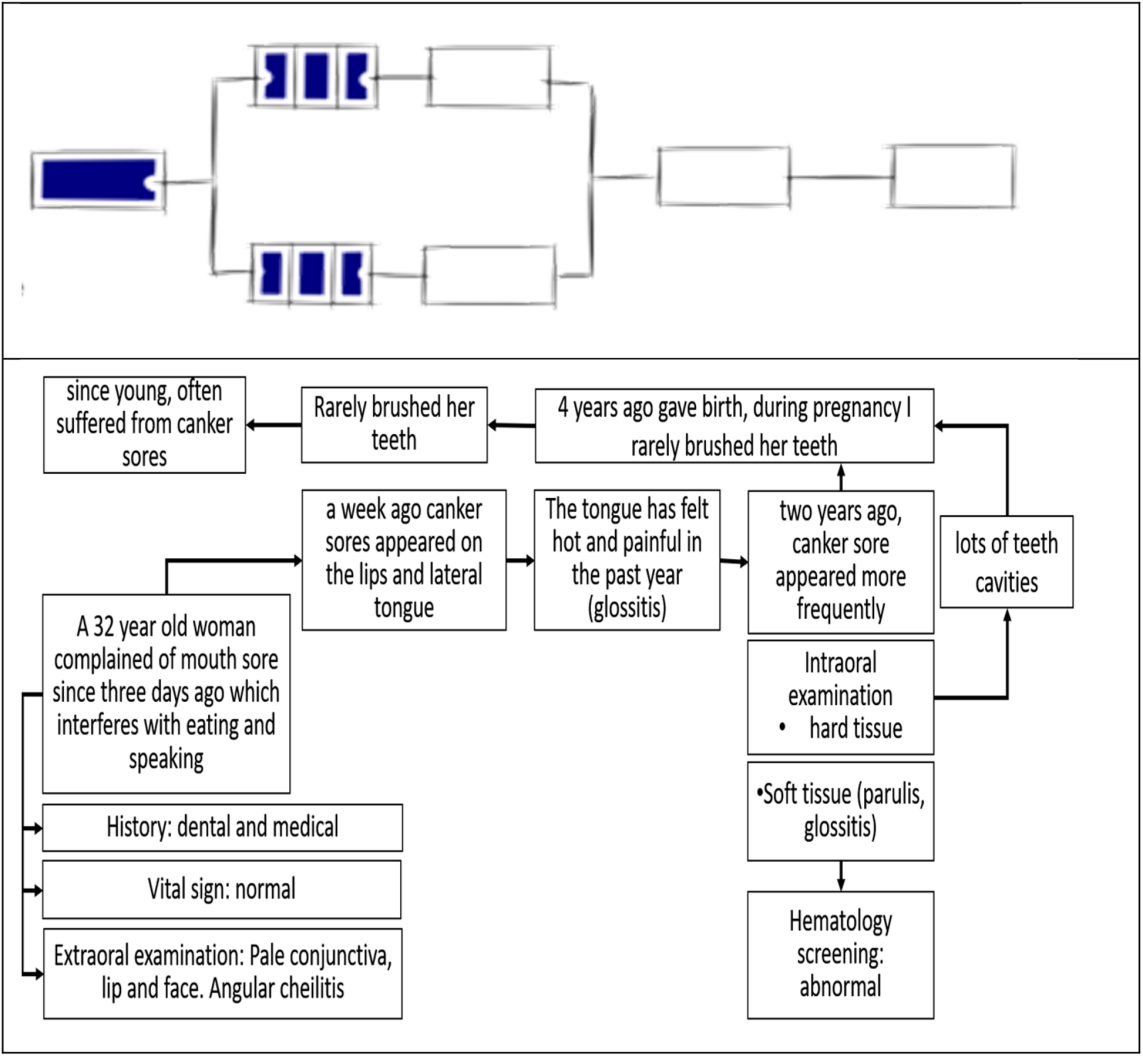
Concept map made by participant #5 supposed to be the time line of patient illnesses, but the thinking approach refers to multistructural knowledge (SOLO taxonomy 3) (The illustrations depicted are from https://pamhook.com/free-resources/ downloadable-resources/)

Participant #5 provided another view to the hypothetical clinical case by stating that the main problem was toothache. Furthermore, new information was provided, but the participant still stated that the possible diagnosis was irreversible pulpitis, which hurts when exposed to stimuli, such as eating. The differential diagnosis were aphthous stomatitis and oral candidiasis, which can cause difficulty in speaking. The participant attempted to compile all clinical information and determine the possible diagnosis. Participants only focused on the clinical findings of the oral cavity and did not relate to one another even to the systemic clinical findings. A hypothetico-deductive model is regarded as a clinical reasoning pattern based on the problem-solving strategy showed by participants. The diagram created by participant does not simply depict any concept map (Fig. 5), because it is close to the disease evolution. The diagram reflected the level of knowledge as the multi-structural level (SOLO taxonomy 3), whose focus on several relevant facts but treated separately.

## Discussion

Clinical reasoning is essential in practice-based disciplines, in the way critical thinking be applied in clinical situations [31]. Clinical reasoning is the main task of clinicians in diagnosis and treatment [38, 66, 67]. Developing clinical reasoning skills is a critical part of a bigger, unified identity that learner will need to bring to clinical experiences to participate in caring for patients and work in teams [66, 67]. Clinical reasoning is required to clinical practice daily; however, it is not directly taught during didactical stage, but is given deliberately through clinical practice [2, 8, 68–70]. Dental education previously emphasized critical thinking during clinical decision making; what, when and how to determine dental procedure for oral complaints. Therefore, the portion of learning biomedical knowledge during didactic stage is less than that of clinical procedural or instrumental knowledge [18, 71, 72]. The Academic Guidelines of Faculty of Dentistry, University of Gadjah Mada, reported that from the total curriculum credits, approximately 20% are dedicated to basic knowledge and the other 20% is for biomedical knowledge along with behavioral science. All the teaching and learning processes occur in the early stage of didactic phase. The teaching and learning process later emphasizes on procedural knowledge and practicing to train psychomotor skills [73, 74].

The clinical findings in hypothetical clinical cases are related to the scope of oral medicine, one of the clinical sciences of dentistry. The initial course is Oral Diagnosis, followed by Diagnosis of Oral Disease, Treatment of Oral Disease, and Dental Management of Medically Compromised Patients. All course requires a good understanding and comprehension of basic science and biomedical knowledge. In addition to routine practicum, educator apply case-based learning to implement “early clinical exposure”. Learners in groups are asked to solve clinical problems from hypothetical cases, recap it as a concept map for easy understanding, and present and conduct a discussion. This condition considered as naturalistic experiment of qualitative studies, which is the commonly research design used for studying clinical reasoning process. Situations, where intervention occurs naturally without planning, refers to naturalistic conditions, resembling experimental requirements [75].

The clinical reasoning is a problem-solving process or strategy which commonly used by clinicians. The effectiveness and efficiency of its application determine how well the knowledge of a clinician when providing patient care, which refers to “expertise” [2, 76]. On acquiring expertise, learners knowledge and skills progress through several transitory stages, as novice or beginner, intermediate to expert, which are characterized by different knowledge structures. The learner gains a considerable amount of basic and biomedical knowledge in the early stage of didactic phase. These concepts are linked together in a knowledge network; additional concept are gradually included and refined and formed a sophisticated knowledge network [13, 42, 49]. The clinical reasoning process can be characterized at every stage of expertise development by a line of reasoning, comprising a chain of small steps based on relevant clinical knowledge. The initial development of the clinical reasoning process is marked by reduced knowledge followed by dispersed, elaborated causal, scheme and script [42]. The chain of steps of knowledge resembles the level of knowledge structure of SOLO taxonomy.

Learners as a novice with a “pre-structural level of knowledge” has scattered bits of information, but in an unorganized structure and minimal comprehension [55]. These description has similarity with “reduced knowledge”, which presented minimal or no knowledge regarding clinical information related to certain disease. Learner with these level of knowledge will applied guessing when solving problem [42]. Chan [64] described “wild guessing” as one of the characteristic for the pre-structural level of the SOLO Taxonomy. This is in accordance with the findings of this study, where participant #1 with prestructural levels and reduced knowledge applied guessing in problem solving. This finding is proven with extended time, more additional questions and statements of nescience shown by participants during the interview. Participant #1 attempted to recall some textbook-related clinical fact, examining available clinical data and trying to match the hypothesis or diagnosis that has been established.

The initial knowledge network is built by the end of the first stage of knowledge acquisition, allowing learner to directly connect the lines of reasoning between different concepts within a network. In this stage, learner will have “dispersed knowledge”, which mean that learners have minimal clinical knowledge [13, 42]. The aforementioned knowledge is similar to “the unistructural level of knowledge”, wherein the learner manifests a correct grasp of one or two relevant pieces of information obtained directly from the problem but lack of appropriate relations to each other [57, 64]. Based on this limited clinical knowledge, the learner uses hypothetical-deductive reasoning during problem solving [42] Participants #3 and #5, both showed to have “dispersed knowledge” and applied a hypothetico-deductive model of clinical reasoning, despite their differences on level of knowledge. These clinical reasoning pattern applied is close to “deductive or backward-oriented reasoning,” where the initial statement is general and moves toward a specific hypotheses, later the addition of new information led to a certain diagnosis [77, 78]. A similar result is found in study by Nafea [22], where deductive or backward reasoning is applied when novice solves clinical problem. The hypothetico-deductive model of clinical reasoning is regarded as characteristic of novice reasoning [39, 79].

The structure of knowledge level 3 (multistructural) SOLO taxonomy can represent end stage of “dispersed knowledge,” while the knowledge structure level 4 (relational) SOLO taxonomy represents as an early stage of “elaborated causal network”. Both structures can show slight connectivity between one or two aspects despite being novice. The multi-structural level of knowledge has an incomplete understanding due to the emphasis on several relevant concepts, recognizing only the relation between these concepts but without further elaboration [57, 64]. Learner with an “elaborated causal network,” has more knowledge regarding diseases with detailed cause-effect links. Learners can explain the causes and consequences of disease considering general underlying pathophysiological process [13, 28]. Participant #2, who showed multistructural level of knowledge with an elaborated causal network applied an ‘elaborated model of hypothetico-deductive reasoning’. Learner reasoned by generating few hypotheses very early as soon as the first pieces of data became available and then testing a set of hypotheses to account for clinical data. The learner selectively collected data focusing only on the relevant data and applied hypothetico-deductive process in the end [80].

Learner with relational level of knowledge (SOLO taxonomy 4) shows an understanding of various concepts and their integration to form a coherent structure [55]. Participant #4 with these level of knowledge applied “scheme-inductive reasoning” when solving the problem. Participant #4 is unique, considering that problem solving strategies through scheme-inductive reasoning patterns are usually found in clinicians with “scheme”, a more sophisticated knowledge level [42], which refers to organized knowledge structure for diagnostic reasoning [63]. Scheme-inductive reasoning pattern begins with establishing several hypotheses that might cause the disease immediately after obtaining initial clinical information. The new information obtained is used to rule out unsuitable hypotheses, add new hypotheses or be combined and analyzed with prior clinical knowledge to sharpen the established hypotheses, until the final diagnosis is established [13, 39, 81].

**Fig. 6:**
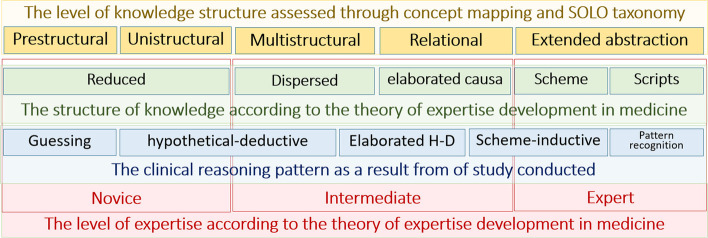
Development of clinical reasoning pattern based on the knowledge structure changes [42]

Fig. 6 summarized the study result which expressed the connection between the clinical reasoning pattern obtained through verbal protocol, the level of knowledge structure assessed through concept map and compared to the SOLO taxonomy, the structure of knowledge and the level of expertise in accordance to theory of expertise development in medicine [42, 82].

Clinical problems solving is a difficult task for under-graduate dental students.

As health professional, they must be able to demonstrate competence in clinical knowledge and skills. Dental undergraduate students were naturally concerned with symptoms and signs as reported by patient or based on written medical records or hypothetical cases due to their limited previous clinical experience with real patients, later recalling and retrieving their knowledge of physical manifestations of diseases from memory [37, 83]. Theoretical conceptual knowledge obtained during didactical phase, which occurs in the first four years, the next two years served as a clinical stage. The teaching and learning processes are largely based on textbook; hence, the resulting perspective on disease is prototypical, with only limited comprehension of the variability of disease manifestations in reality [39]. Prototype refers to organization of clinical categories in memory around particular exemplars and serve as anchors for other members of the category [84]. By contrast, clinical procedural knowledge taught during didactical phase is limited to theory and simulation during practicum [42, 85]. Therefore, only some characteristics of the knowledge structure from the stages of clinical reasoning development were found in this study, knowledge structure as “scheme” and “script” were absent. Both of knowledge structure were developed when a learner has increased their expertise.

This study revealed the unique findings, which can be intended as the weaknesses or the strengths of the study. The weakness of the study involves some qualitative studies characteristics and limitation, including sampling technique, number of participants, characteristic of participants, no control over variables and confounding factors, specificity of hypothetical case, data acquisition, and data interpretating consistency. Some of these points can lead to research bias. Although there are no strict requirements on the sampling technique and number of participants as quantitative study, the selection of the sampling technique and the determination of the number of participants have been determined in such a way that the results of the study will be in accordance with the research aims. The main weakness of the findings is not being able to generalize, because the relevance of this findings is aimed at providing information and suggestions for improvement for students and dental institutions involved. Moreover, the hypothetical case shows, the specificity of the observations on certain knowledge. In order to be relevant for another disciplines, it is necessary to modify the hypothetical case script.

The strength of this study is the finding of knowledge gaps which refer to the discrepancy between the level of expertise with structure of knowledge as well as pattern of clinical reasoning, as shown on Fig. 6. Undergraduate dental students as novices, with the lowest level of expertise according to the theory of expertise development in medicine [42]. As a novice, the knowledge acquisition shows the lowest level and structure of knowledge, that will affect problem solving strategies. But in this study, the participants who were all undergraduate dental students showed varying levels and structures of knowledge, even clinical reasoning patterns that did not match their level of expertise. The theory of development of medical expertise cannot be precisely applied to the dentistry, even though this study employs the knowledge of oral medicine which is similar to medical knowledge. Further observations are needed regarding these issues.

## Conclusion

Various problem-solving strategies were encountered in this study, ranging from guessing to sophisticated clinical reasoning patterns, which corresponded to the level of knowledge acquisition. There are three strategies used to solve clinical problems identified in this study, which each is dependent on the evolution of the structure or level of knowledge, comprising the following: guessing based on reduced knowledge or prestructural level of knowledge; hypothetical deductive reasoning based on dispersed knowledge or unistructural level of knowledge, elaborated hypothetical deductive reasoning based on elaborated causal knowledge or multistructural level of knowledge; and scheme inductive reasoning based on schemed knowledge or relational level of knowledge. Despite no identified in this study, there is an expertise strategy for clinical problem solving namely pattern recognition, which is based on scripted knowledge or extended abstraction level of knowledge.

## Recommendations

Dental institutions must set a minimum standard regarding the acquisition of conceptual knowledge along with improving clinical reasoning skills, refining the procedural knowledge and skills. Thus, improving dental education, including curriculum, teaching and learning methods, instructional methods, or dental environment, which emphasize clinical reasoning to provide optimal dental health services is suggested.

## Supplementary Information

**Additional file 1.**

**Additional file 2.**

**Additional file 3.**

## Data Availability

The data presented in this study are available upon request from the corresponding author. Complete written data are not provided for publication due to language barriers (the language used is Bahasa Indonesia, which is the native language of the participants and the authors). However, the original concept maps drawn by all participants were presented in Appendix C, due to the universality of graphs.
