# Supplementary material for: Clinical reasoning pattern used in oral health problem solving – A case study in Indonesian undergraduate dental students
Source: BMC Med Educ. 2023 Jan 23;23:52. doi: 10.1186/s12909-022-03808-7 (PMC9872386; doi:10.1186/s12909-022-03808-7)
Supplement: Supplementary file 1 — Additional file 1. [file 12909_2022_3808_MOESM1_ESM.docx]

**Appendix A. Hypothetical clinical case as a trigger for clinical problem-solving**

The oral health problem scenario describes a 32-year-old woman complaining sore mouth in the past three days, which interferes with eating and talking. She is suffering from recurrent oral ulcers since her youth; however, these ulcers develop and worsened as she got old. She is also complaining a burning sensation on her tongue and painful crack at both corners of the mouth. Medical history revealed that she has rarely eaten fruits and avoided red meat and milk since adolescence and also experiences heavy menstruation accompanied by abdominal pain. She gave birth to twins four years ago and took care of them by herself. General examination indicated the following: height is 160 cm, weight is 45 kg, BP is 110/70 mm. hg, and pain scale is 5. Her face, lips and conjunctiva look pale on extraoral examination. The lips were dry and some peeled off, both corners were cracked and inflamed. Intraoral examination showed tooth #47 severely caries with sharp edges on buccal side, radices #36 and moderate gingivitis in the upper and lower anterior regions. Yellowish nodule Ø 4 mm were found at gingiva as high as the apex of tooth # 36, with smooth texture and soft consistency. The right buccal mucosa opposite the molar teeth # 47 showed a single ulcer Ø 8 mm with an oval shape, irregular and edematous margin covered with a whitish pseudomembranous, surrounded by erythematous halo. The radiographic finding was a radiolucent area Ø 3mm with obscure border at mesial root tooth #36. The blood tests revealed 92 mg/dL hemoglobin (Hb) and 28,4% hematocrit (Hct).
