# Supplementary material for: Clinical reasoning pattern used in oral health problem solving – A case study in Indonesian undergraduate dental students
Source: BMC Med Educ. 2023 Jan 23;23:52. doi: 10.1186/s12909-022-03808-7 (PMC9872386; doi:10.1186/s12909-022-03808-7)
Supplement: Supplementary file 2 — Additional file 2. [file 12909_2022_3808_MOESM2_ESM.docx]

**Appendix B. Interpretation of hypothetical clinical case**

Clinical-problem solving in dentistry generally leads to treatment options or procedural skills, while oral medicine emphasis on diagnostic skills which later lead to treatment. These skills resemble problem-solving in medicine. The clinical reasoning process comprises the following: a) identification of the relevant clinical information to diagnosis; b) understanding of its meaning; c) generation of hypotheses or probable diagnosis which are coherent the patient’s problem’s; d) testing and refining hypotheses through further data gathering and e) establishment of a working or most probable diagnosis. The steps for establishing a differential diagnosis of oral lesion as follows: a) categorize the oral abnormality; 2) gather secondary data; 3) determine several possible causes; 4) rule out improbable etiology; 5) make a differential diagnosis; and 6) set a working diagnosis.

In the aforementioned hypothetical case, steps to solve the problem can be arranged as follows.

1. The clinical problem in the case is “mouth sore” that affects oral function, which can be distinguished as odontogenic, mucosal or neurological pain. Referring to the history, pain is caused by mucosal disintegration, which can be erosive, atrophic or ulcerous lesions.
2. Relevant secondary data included a history of lesions i.e., involvement of the buccal mucosa and lateral tongue, and disease nature as recurrence, juvenile onset, short duration, self-healing, related to menstrual period and amount of blood, mood, stress, fatigue and workload. Diet history led to suspicion of vitamin deficiency. The clinical findings that must be considered include the location, shape, number and clinical characteristic of the lesion, the presence of possible factors that trigger the lesion (sharp tooth edges). Involvement of other mucosal or body systems accompanying the complaint, such as facial, conjunctiva, lips and mucosal pallor, sores at the corners of the mouth, and the abnormality of the dorsal tongues hould also considered. The results of the hematological test indicated the presence of an anemic condition. The other clinical data might be irrelevant to main problem.
3. Various pathological conditions are reported to have similar clinical features, such as reactive, infection, genetic/hereditary condition or disease, inflammation, hypersensitivity or immune reaction, auto-immune disease, malignancies, oral manifestations of systemic diseases, part of syndromes and even idiopathic conditions.
4. The possible etiology considering primary manifestations, secondary clinical findings including additional investigations, revealed the following: reactive led to inflammation, hormonal related to the hereditary condition and oral manifestations of systemic disease.
5. The differential diagnosis of the main complaint includes traumatic ulceration due to sharp tooth edges, recurrent aphthous stomatitis and aphthous-like ulcer related to anemia and nutrition deficiency.
6. Determination of a working diagnosis is important because it focuses on the priority of treatment. In this case, sharp tooth edges cannot be ignored because they can which can exacerbate the severity oral ulcers and the systemic background does not support the appropriate healing process. Therefore, a traumatic ulcer is regarde as the initial working diagnosis, and eliminating the causative tooth is expected to accelerate healing. Along with the replacement therapy for anemia, nutritional deficiencies must be addressed followed by improvement in nutritional intake. The subsequent dental procedure depends on the priority of treatment set by the dentist as elective treatment, which can include extraction for other pathological teeth, root canal treatment or periodontal treatment.
