# Supplementary material for: Clinical reasoning pattern used in oral health problem solving – A case study in Indonesian undergraduate dental students
Source: BMC Med Educ. 2023 Jan 23;23:52. doi: 10.1186/s12909-022-03808-7 (PMC9872386; doi:10.1186/s12909-022-03808-7)
Supplement: Supplementary file 3 — Additional file 3. [file 12909_2022_3808_MOESM3_ESM.docx]

**Appendix C. Original graph represents the clinical concept map drawn by participant**

Concept map by participant #1

| 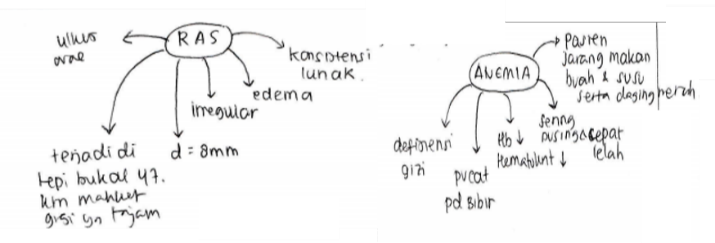 |
| --- |

Concept map by participant #2

| 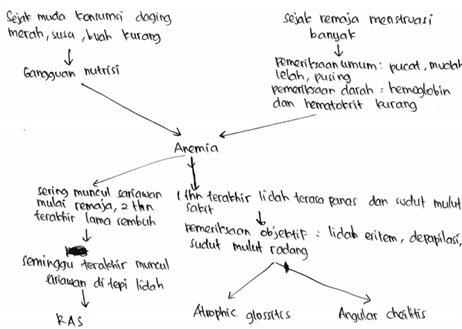 |
| --- |

Concept map by participant #3

| 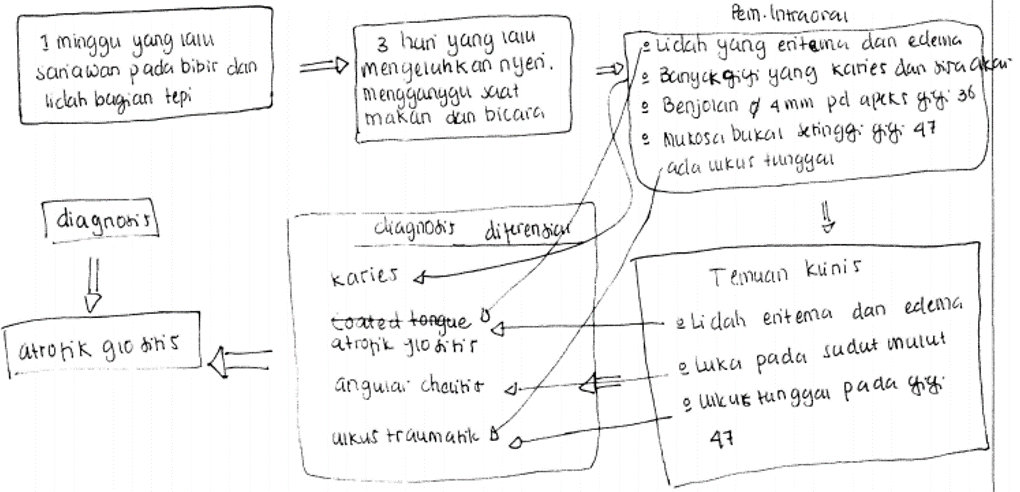 |
| --- |

Concept map by participant #4

| 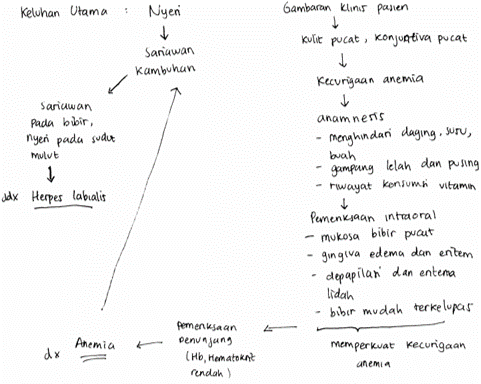 |
| --- |

Concept map by participant #5

| 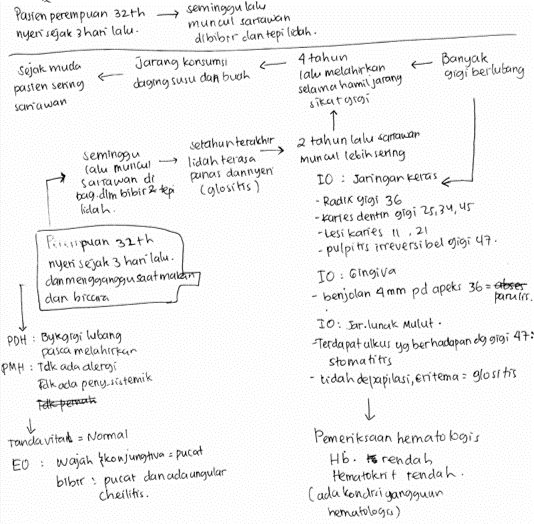 |
| --- |
